# *Pseudomonas aeruginosa* *mexR* and *mexEF* Antibiotic Efflux Pump Variants Exhibit Increased Virulence

**DOI:** 10.3390/antibiotics10101164

**Published:** 2021-09-25

**Authors:** Mylene Vaillancourt, Sam P. Limsuwannarot, Catherine Bresee, Rahgavi Poopalarajah, Peter Jorth

**Affiliations:** 1 Department of Pathology and Laboratory Medicine, Cedars-Sinai Medical Center, Los Angeles, CA 90048, USA; Mylene.Vaillancourt@cshs.org (M.V.); Podsawee.Limsuwannarot@cshs.org (S.P.L.); 2 Biostatistics Core, Cedars-Sinai Medical Center, Los Angeles, CA 90048, USA; Catherine.Bresee@cshs.org; 3 Department of Biological Sciences, University of Calgary, Calgary, AB T2N 1N4, Canada; Rahgavi.Poopalarajah@ucalgary.ca; 4 Department of Medicine, Cedars-Sinai Medical Center, Los Angeles, CA 90048, USA; 5 Department of Biomedical Sciences, Cedars-Sinai Medical Center, Los Angeles, CA 90048, USA

**Keywords:** *Pseudomonas*, efflux pumps, virulence, evolution, antibiotic resistance, cystic fibrosis

## Abstract

Antibiotic-resistant *Pseudomonas aeruginosa* infections are the primary cause of mortality in people with cystic fibrosis (CF). Yet, it has only recently become appreciated that resistance mutations can also increase *P. aeruginosa* virulence, even in the absence of antibiotics. Moreover, the mechanisms by which resistance mutations increase virulence are poorly understood. In this study we tested the hypothesis that mutations affecting efflux pumps can directly increase *P. aeruginosa* virulence. Using genetics, physiological assays, and model infections, we show that efflux pump mutations can increase virulence. Mutations of the *mexEF* efflux pump system increased swarming, rhamnolipid production, and lethality in a mouse infection model, while mutations in *mexR* that increased expression of the *mexAB-oprM* efflux system increased virulence during an acute murine lung infection without affecting swarming or rhamnolipid gene expression. Finally, we show that an efflux pump inhibitor, which represents a proposed novel treatment approach for *P. aeruginosa*, increased rhamnolipid gene expression in a dose-dependent manner. This finding is important because rhamnolipids are key virulence factors involved in dissemination through epithelial barriers and cause neutrophil necrosis. Together, these data show how current and proposed future anti-Pseudomonal treatments may unintentionally make infections worse by increasing virulence. Therefore, treatments that target efflux should be pursued with caution.

## 1. Introduction

*Pseudomonas aeruginosa* is a ubiquitous Gram-negative bacterium that colonizes a wide range of environments [1]. This environmental flexibility is due to its high adaptability to changing conditions and is driven by substantial metabolic versatility, expression of a large array of virulence factors, and extensive adaptive transport and efflux systems [2]. *P. aeruginosa* is infamous for causing serious nosocomial infections, such as burn wound infections and ventilator-associated pneumonia [3], and it is the predominant pathogen causing chronic infections in cystic fibrosis (CF) [4].

During chronic infections, *P. aeruginosa* adapts to the CF lung environment by decreasing production of virulence factors and evolves to become antibiotic resistant [5,6,7]. Even more complicated, populations of *P. aeruginosa* within patients evolve high degrees of diversity and within-patient variability, where contemporary sibling cells that have descended from a common ancestor can differ in antibiotic susceptibilities and virulence factor production [8,9,10,11]. *P. aeruginosa* virulence mechanisms include attachment to cells (e.g., pili), induction of host damage (e.g., DNase, proteases), competition with the host for nutrients, and the secretion of surfactant molecules such as rhamnolipids which cause neutrophil necrosis and permit dissemination [12,13,14]. Virulence factors usually trigger an exacerbated inflammatory response by the innate immune system, with macrophages and neutrophils as the first line of defense [15]. Thus, it has been proposed that decreasing virulence factor production attenuates the host inflammatory response and favors bacterial colonization of the lung during CF chronic infections.

In addition to the host pressures that affect virulence, bacteria colonizing CF lungs also experience intense antibiotic pressure due to mono and combination therapies including aminoglycosides, quinolones, and β-lactams [16]. This environment strongly favors the selection of antibiotic-resistant strains. Some of the best characterized antibiotic resistance features are efflux pump systems that expel antibiotics out of the cell. For example, we previously showed that *nalD* and *mexR* genes, both encoding for transcriptional repressors of the MexAB-OprM efflux pump [17,18,19], were recurrently mutated under aztreonam selection [20]. *P. aeruginosa* with mutations in *nalD* and *mexR* overexpress the MexAB-OprM efflux pump, rendering them multidrug resistant [17,19,21]. Moreover, mutations in these two genes have been found in anywhere from 7–47% of clinical isolates in various studies [20,21,22,23,24]. Importantly, strains with mutations in *nalD* and *mexR* evolved under aztreonam selection showed increased virulence *in vivo*, challenging the longstanding theory that antibiotic-resistance mutations inherently pay fitness costs that attenuate virulence [20]. However, whether these mutations increase virulence in an efflux-dependent manner remains unknown.

In this study, we sought to investigate the mechanisms involved in the hypervirulence of a mutant *P. aeruginosa* strain, PAO1-AzEvB8, that was experimentally evolved in a previous study during cyclic aztreonam exposure [20]. Because this strain was previously shown to have a mutation in *mexR*, we hypothesized that its hypervirulence could be attributed to the increased expression of the MexAB-OprM efflux pump. We explore this hypothesis using a combination of genome sequencing, genetics, transcriptional reporter assays, and infection models.

## 2. Results

### 2.1. Deletion of mexAB Restores Aztreonam Susceptibility of Evolved P. aeruginosa

**Figure 1:**
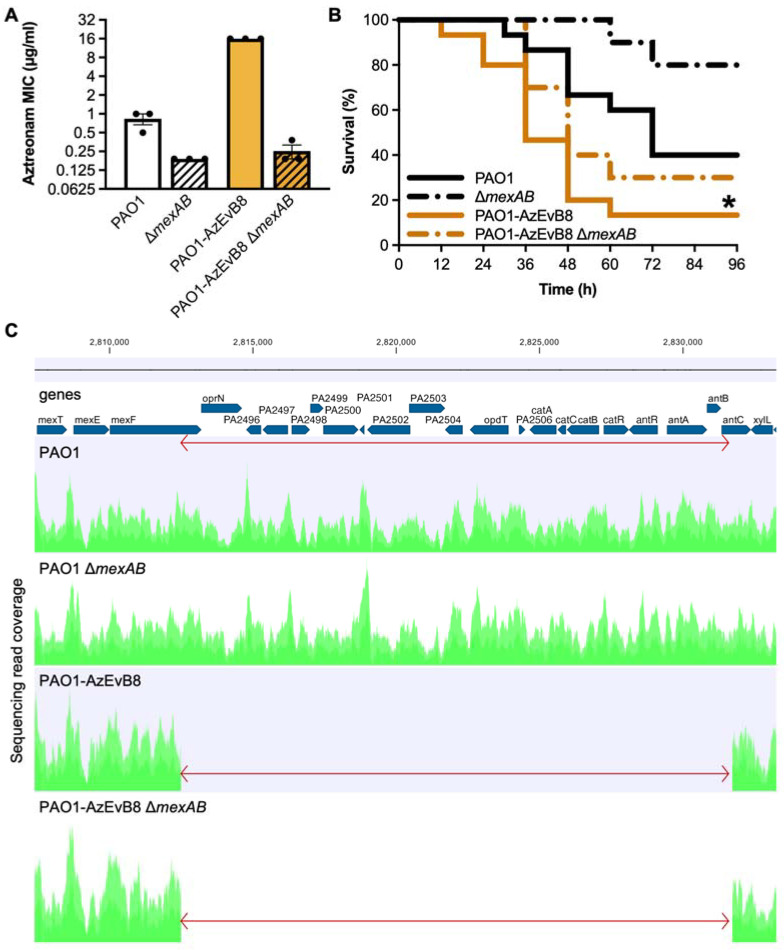
MexAB-OprM is not involved in the PAO1-AzEvB8 mutant in vivo virulence. (**A**) Deletion of the *mexAB* genes abrogates aztreonam resistance in strain PAO1-AzEvB8. Aztreonam MICs were determined by gradient diffusion assays (n = 3 replicates/group; MIC: minimum inhibitory concentration). (**B**) *mexAB* deletion in PAO1-AzEvB8 mutant does not significantly improve mouse survival following acute lung infection. Log-rank test was used to compare survival curves. * *p* < 0.05 compared to WT PAO1; n = 10–15 mice/group. (**C**) Genome diagram showing coverage of sequencing reads aligning to the region spanning *mexEF* through *xylZ*. The 19,233 bp deleted region (indicated by the red arrow) in strains PAO1-AzEvB8 and PAO1-AzEvB8 Δ*mexAB* begins at the 3′ end of *mexF* and continues through the 5′ region of *antC*. Genome coverage plots generated from sequencing read alignments to the PAO1 reference genome are indicated in green.

Previously, we used experimental evolution with cyclical exposure to aztreonam to evolve the aztreonam resistant strain PAO1-AzEvB8 [20]. Strain PAO1-AzEvB8 was found to have a nonsense mutation in *mexR* (E118*) and complementation with a wild-type (WT) *mexR* gene restored aztreonam susceptibility [20]. Because *mexR* mutations have been shown to increase expression of the *mexAB-oprM* efflux pump operon [19,21], which encodes the pump that effluxes aztreonam, we hypothesized that deletion of *mexAB* in PAO1-AzEvB8 would restore aztreonam susceptibility. We generated clean *mexAB* deletions in both WT PAO1 and in PAO1-AzEvB8, and the PAO1-AzEvB8 Δ*mexAB* strain was equally susceptible to aztreonam by a gradient diffusion assay as the PAO1 Δ*mexAB* strain (Figure 1A); the mode minimum inhibitory concentrations (MIC) for Δ*mexAB* strains was 0.19 μg/mL, compared to 16 μg/mL for PAO1-AzEvB8 and 1.0 μg/mL for WT PAO1. This showed that *mexAB* was required for aztreonam resistance of the of the PAO1-AzEvB8 mutant.

### 2.2. Deletion of mexAB Does Not Affect Virulence of Evolved P. aeruginosa

In addition to exhibiting increased aztreonam resistance relative to WT PAO1, strain PAO1-AzEvB8 also displayed increased killing of mice in an acute murine infection model. We hypothesized that, similar to the aztreonam resistance, the increased virulence of PAO1-AzEvB8 would be dependent upon *mexAB* which was overexpressed in this strain. Surprisingly, the PAO1-AzEvB8 Δ*mexAB* strain was equally virulent to strain PAO1-AzEvB8 (Figure 1B), indicating that the increased virulence was not dependent on *mexAB* alone. This was even more unexpected since PAO1 Δ*mexAB* was less virulent than WT PAO1 (Figure 1B). This suggested that another gene or mutation was involved in the enhanced virulence of PAO1-AzEvB8.

### 2.3. Genome Sequencing Reveals a Previously Undetected 19 kb Deletion in PAO1-AzEvB8

To determine whether another mutation may be present in the strain PAO1-AzEvB8, we performed whole genome sequencing on strains PAO1 (WT parent strain), PAO1 Δ*mexAB*, PAO1-AzEvB8, and PAO1-AzEvB8 Δ*mexAB*. Using this approach, we found that relative to WT PAO1, a large ~19 kb deletion affecting 21 genes spanning *mexF* through *antC* was present in the PAO1-AzEvB8 and PAO1-AzEvB8 Δ*mexAB* strains (Figure 1C). This deletion was not detected by the genome sequencing in our original study despite >10-fold sequencing coverage of the genome [20]. The genes affected by this mutation included *mexF* and *oprN* which form part of the *mexEF-oprN* efflux pump operon. Previously, these genes have been linked to the swarming phenotype in *P. aeruginosa*, with studies indicating that strains overexpressing *mexE* have decreased swarming [25,26]. This led us to predict that the *mexF* mutation could lead to increased swarming and rhamnolipid production in the PAO1-AzEvB8 strain.

### 2.4. Mutation of mexEF Increases Swarming While mexR Mutation Does not

**Figure 2:**
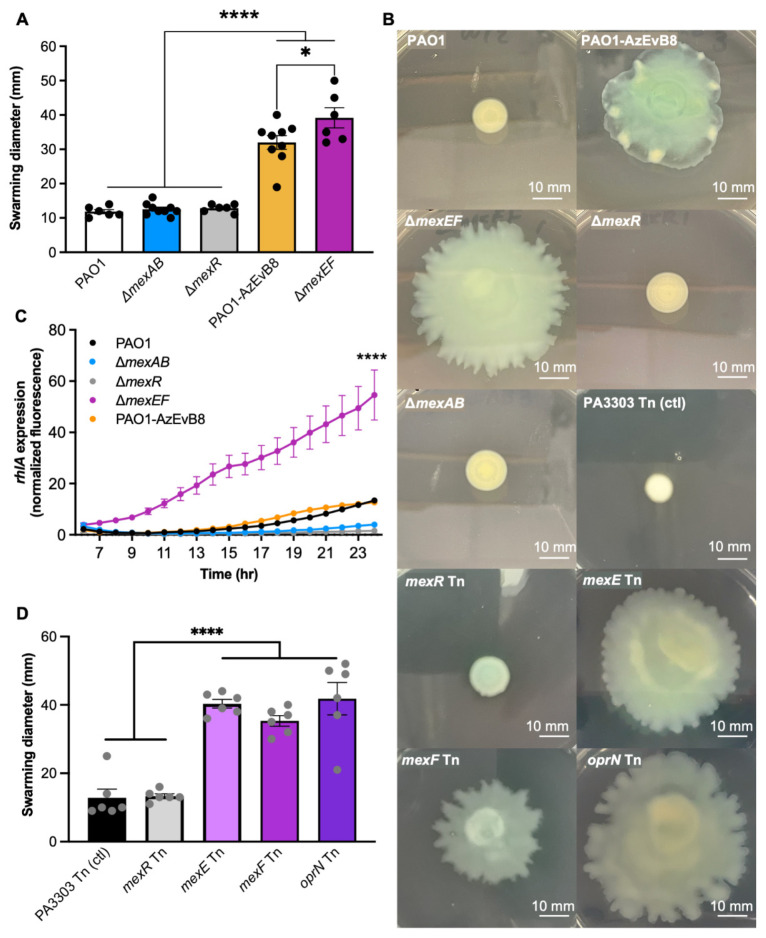
MexEF-OprN mutation increases swarming motility and biosurfactant production. (**A**) Swarming is increased in the PAO1-AzEvB8 and Δ*mexEF* strains. Measurement of the swarming motility in different *mexAB-oprM* and *mexEF-oprN* mutants after 24 h, n = 6–7 replicates/group. (**B**) Representative images of swarming motility from panels (**A**,**D**). Scale bars indicate 10 mm. (**C**) Rhamnolipid gene expression is increased in the Δ*mexEF* strain. Rhamnolipid production measured by quantification of *rhlA* gene expression using a *rhlA*-*gfp* promoter reporter fusion to measure GFP fluorescence over time. Gene expression was calculated as area under the curve for each strain and compared to all other groups, n = 6–7 replicates/group. (**D**) Swarming is increased in *mexE, mexF,* and *oprN* PAO1 transposon mutant strains. Measurement of the swarming motility in different *mexR* and *mexEF-oprN* transposon mutants vs. a neutral Tn mutant control (PA3033) after 24 h, n = 5–12 replicates/group. For all panels, * *p* < 0.05, **** *p* < 0.0001, one-way ANOVA, followed by a Tukey’s multiple comparisons test.

Based on the deletion of a large part of the *mexEF-oprN* operon in strain PAO1-AzEvB8, we hypothesized that this strain would exhibit increased swarming, as would mutants lacking *mexEF*. To test this, we generated a strain with the *mexEF* genes deleted in a WT PAO1 background. Gradient diffusion susceptibility testing showed that the PAO1 Δ*mexEF* strain was more susceptible to both ciprofloxacin and chloramphenicol than WT PAO1 (mode ciprofloxacin MICs: PAO1 Δ*mexEF* 0.125 μg/mL vs. WT PAO1 0.5 μg/mL; mode chloramphenicol MICs: PAO1 Δ*mexEF* 48 μg/mL vs. WT PAO1 >256 μg/mL; n = 3). Swarming assays were performed using the WT PAO1, PAO1-AzEvB8, PAO1 Δ*mexEF*, and PAO1 Δ*mexR* strains. As predicted, the PAO1-AzEvB8 strain displayed more swarming than WT, as did PAO1 Δ*mexEF* (Figure 2A,B). In contrast, deletion of *mexR* in a WT PAO1 background did not affect swarming (Figure 2A,B). Next, we tested whether *mexEF* mutations led to increased rhamnolipid production, because rhamnolipids are involved in swarming and cause necrosis of neutrophils [13]. A *rhlA*-*gfp* transcriptional reporter was transformed into strains PAO1, PAO1 Δ*mexEF*, PAO1 Δ*mexAB*, PAO1 Δ*mexR*, and PAO1-AzEvB8 and *rhlA* expression was monitored during the course of growth. Consistent with the swarming phenotypes, PAO1 Δ*mexEF* had the highest *rhlA* expression (Figure 2C). However, unexpectedly, PAO1-AzEvB8 had less *rhlA* expression than PAO1 Δ*mexEF*, similar *rhlA* expression to WT PAO1, and slightly more *rhlA* expression than PAO1 Δ*mexAB* and PAO1 Δ*mexR* strains (Figure 2C). Together these data showed that the PAO1-AzEvB8 strain had a hyper-swarming phenotype, a *mexEF* deletion mutation was sufficient to increase swarming, and a *mexR* deletion mutation alone does not affect swarming.

To further study the effects of *mexEF* mutations on swarming and rhamnolipid gene expression, we also investigated these phenotypes using transposon (Tn) mutants. Swarming assays showed the PAO1 Tn mutants in *mexE, mexF,* and *oprN* all exhibited increased swarming relative to a Tn mutant control (PA3033), which has been shown to be a neutral Tn mutation for numerous phenotypes. As above, a *mexR* Tn mutant showed the same levels of swarming as the Tn mutant control.

### 2.5. Deletion of either mexR or mexEF Increases P. aeruginosa Virulence

**Figure 3:**
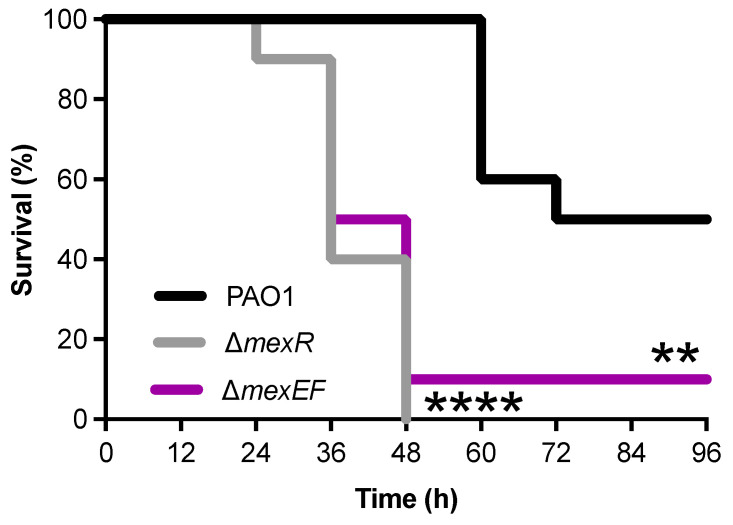
MexAB-OprM overexpression and MexEF-OprN deletion exhibit increased in vivo virulence. Both Δ*mexR and* Δ*mexEF* mutations in WT PAO1 significantly reduce mouse survival during an acute lung infection compared to WT PAO1 (WT). Survival times were tested across strata with a Bonferroni-adjusted log-rank test: ** *p* < 0.005 and **** *p* < 0.0001 compared to WT PAO1, n = 10 mice/group.

To better understand the increased virulence of the PAO1-AzEvB8 strain, we analyzed the virulence of PAO1 Δ*mexR* and PAO1 Δ*mexEF* strains to determine if either the *mexR* or *mexF* mutations detected in the PAO1-AzEvB8 strain could have contributed to the increased virulence. Using an acute murine lung infection model, we found that both PAO1 Δ*mexR* and PAO1 Δ*mexEF* killed the infected mice significantly faster than the WT PAO1 strain (Figure 3). These findings suggest that either of the two mutations present in the PAO1-AzEvB8 were sufficient to increase the virulence, relative to the WT parent strain.

### 2.6. Efflux Pump Inhibition Increases Rhamnolipid Virulence Factor Expression

**Figure 4:**
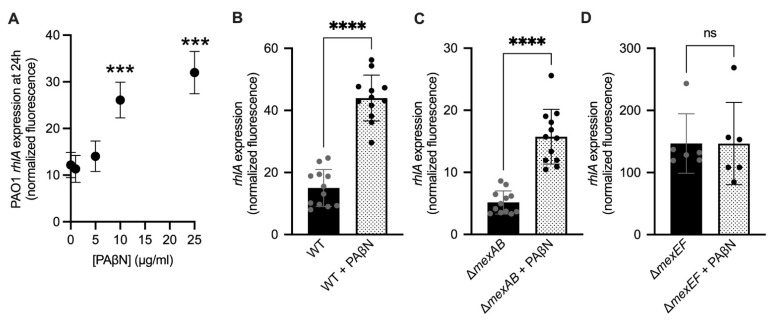
Rhamnolipid gene expression is induced by the PaβN efflux pump inhibitor. **(A)** *rhlA* expression in WT PAO1 *attB::rhlA-gfp* treated with 0–25 μg/mL PAβN as measured by GFP fluorescence from the *attB::rhlA-gfp* promoter reporter fusion at 24 h (*** *p* < 0.0005, compared 0, 1, and 5 μg/mL, one-way Brown−Forsythe and Welch ANOVA followed by Dunnett’s T3 multiple comparisons test, n= 6 replicates/group). (**B**–**D**) *rhlA* expression in PAO1 *attB::rhlA-gfp* (**B**), PAO1 Δ*mexAB attB::rhlA-gfp* (**C**), and PAO1 Δ*mexEF attB::rhlA-gfp* (**D**), treated with or without 25 μg/mL PAβN as measured by GFP fluorescence from the *attB::rhlA-gfp* promoter reporter fusion at 24 h. Gene expression was calculated as GFP fluorescence normalized to cell density (**** *p* < 0.0001; ns: not significant, *p* > 0.05, one-way Brown−Forsythe and Welch ANOVA followed by Dunnett’s T3 multiple comparisons test, n = 6–12 replicates/group).

The swarming assays and mouse virulence experiment suggested that loss of the *mexEF* efflux pump was sufficient to increase swarming, rhamnolipid expression, and virulence in a mouse lung infection model. Therefore, we hypothesized that inhibition of efflux pump activity would lead to increased rhamnolipid gene expression. To test this, we exposed the PAO1 *attB::rhlA*-*gfp* reporter strain to increasing concentrations of the PAβN efflux pump inhibitor [27]. As hypothesized, increasing concentrations of PAβN from 0–25 μg/mL led to increased *rhlA* rhamnolipid gene expression in a dose-dependent manner (Figure 4A). This showed that a broad-spectrum efflux pump inhibitor could increase the expression of an important virulence factor.

Because PAβN can inhibit both MexEF-OprN and MexAB-OprM, we tested the hypothesis that the PAβN inhibitor was increasing rhamnolipid gene expression in a *mexEF*-dependent manner. We incubated WT PAO1, PAO1 Δ*mexAB*, and PAO1 Δ*mexEF rhlA-gfp* reporter strains with or without 25 μg/mL PAβN for 24 h. As predicted, PAβN increased *rhlA* gene expression in WT PAO1 and PAO1 Δ*mexAB* strains relative to untreated controls (Figure 4A–C), but not in the PAO1 Δ*mexEF* strain (Figure 4D). This shows that the increase of *rhlA* expression is occurring via inhibition of MexEF-OprN, not MexAB-OprM.

## 3. Discussion

### 3.1. Deletion of mexEF and Overexpression of mexAB through mexR Mutation Can Each Increase Virulence of P. aeruginosa

Here we showed that two different mutations affecting efflux pumps that evolved in response to aztreonam selective pressure can increase *P. aeruginosa* virulence in the absence of antibiotic treatment. These findings are interesting because one mutation, *mexR*, leads to overexpression of the MexAB-OprM efflux pump, while the other mutation conferred the loss-of-function of a second efflux pump, *mexEF*-*oprN*. We also showed that deletion of *mexEF* caused increased swarming, likely due to increased *rhlA* gene expression, which was demonstrated using a rhamnolipid gene expression reporter. Finally, we showed that MexEF-OprN efflux pump inhibition can have the unexpected effect of increasing rhamnolipid gene expression. This observation is important because rhamnolipids can cause necrosis of neutrophils and are also involved in the penetration of *P. aeruginosa* through epithelial barriers [13,14]. Altogether, these data highlight how antibiotic selection can unexpectedly increase virulence through multiple pathways.

### 3.2. Understanding Why rhlA Was Not Expressed in the PAO1-AzEvB8 Strain That Exhibited Greater Swarming Than WT

One curious result was that the PAO1-AzEvB8 strain did not overexpress *rhlA* relative to WT PAO1, despite exhibiting increased swarming. Because this strain had a large deletion mutation that included the 3′ end of *mexF*, we expected it to exhibit elevated *rhlA* expression similar to the PAO1 Δ*mexEF* strain. One possible explanation is that with an intact *mexE* gene, the increase in *rhlA* relative to WT may be less pronounced than when both *mexE and mexF* are deleted. This is somewhat apparent in the quantification of the swarming assays where the swarming zone of PAO1 Δ*mexEF* was slightly larger than PAO1-AzEvB8. Another possible explanation is that the swarming assays were performed using semi-solid 0.5% agar plates, whereas the *rhlA* expression assays were done in broth cultures. In future work we plan to explore whether differences in *rhlA* expression are detectable during infection, which is where it is likely most relevant.

### 3.3. Relationship of These Findings to Previous Research

Overall, these results are consistent with previous studies which found that *P. aeruginosa nfxC* mutants, which overexpress *mexEF-oprN,* do not swarm and exhibit decreased *rhlA* expression relative to WT [26]. Likewise, these data also align with a study by Cosson et al. which found that *P. aeruginosa nfxC* mutants were avirulent in a rat acute pneumonia infection model, and virulence could be partially restored in the *nfxC* mutant by deleting *mexE* [28]. An important distinction is that, to our knowledge, it had not previously been shown that deletion of *mexEF* in a WT genetic background can increase virulence.

### 3.4. Differences among Infection Models Help Explain Why Previous Studies Concluded Different Effects of Efflux Pump Inhibition on Virulence

While our data agree with the studies discussed above, the finding that the Δ*mexEF* strain was more virulent than WT in the acute murine lung infection does appear to conflict other previous research related to efflux pump inhibition. In a study from Hirakata et al., PAβN was shown to reduce the intracellular invasion of Madin−Darby canine kidney epithelial cell monolayers by *P. aeruginosa* [29], which appears inconsistent with our findings. One simple explanation for this difference is that we were not measuring intracellular invasion in our model. Instead, we measured virulence by survival following acute pneumonia, which we argue is more similar to human lung infections because neutrophils and epithelial barriers are present that can be harmed by the excess rhamnolipid produced in *mexEF* mutants and bacteria treated with PAβN. In another study, PAβN reduced the swarming and killing of *Galleria mellonella* larvae [30]. Again, slight differences in the infection model and the swarming assay could help explain differences from the present study. For example, the medium used for the swarming assay is different from the present study [30]. The *G. mellonella* infection model is also quite distinct from the mouse infection model used here, as it seems to be primarily affected by type III secretion [31] and lipopolysaccharide [32], and the role of rhamnolipids in the *G. mellonella* model is less clear. Therefore, we believe that differences in experimental design can account for the differences in interpretation of the effects of the PAβN efflux pump inhibitor. Future experiments will be required to determine whether PAβN and similar efflux pump inhibitors enhance or decrease virulence in mammalian infections.

### 3.5. Relevance of mexEF Mutations in P. aeruginosa Clinical Isolates

While *mexR* mutations have been identified in countless *P. aeruginosa* clinical isolates in previous studies [24,33], mutations directly affecting *mexEF* have not been as commonly described. Previous work has shown that mutations in *mexT*, the transcriptional activator of *mexEF*, are very common and these mutants were dubbed *nfxC-*type mutants for their resistance to norfloxacin [34,35]. Additionally, several studies have also examined *mexE* gene expression in large sets of clinical isolates, and in some strains *mexE* expression was reported to be less than WT, or completely undetected [22,36,37]. This suggests that these strains have either lost function of *mexT* or may potentially have deletions in *mexE*. This raises the possibility that these clinical isolates would also exhibit increased swarming, increased rhamnolipid production, and be more virulent than other clinical isolates, though this has not yet been investigated. We plan to test these phenotypes in clinical isolates with reduced *mexE* expression in the future.

### 3.6. Implications for CF and the Use of Efflux Pump Inhibitor as Potential Therapies

These results have several implications that are particularly relevant to CF treatment, including currently used anti-Pseudomonal therapies, as well as proposed new therapies.

First, these data show how aztreonam, a common antibiotic used to treat *P. aeruginosa* infections in people with CF, can select for not one, but two different mutations that increase *P. aeruginosa* virulence. This may help explain why a recent clinical observational study found that people with CF that were infected with aztreonam-resistant *P. aeruginosa* were more likely to experience pulmonary exacerbations and be hospitalized than people infected with aztreonam-susceptible *P. aeruginosa* [38]. If the isolates in those patients evolved mutations in either *mexR* or *mexEF-oprN*, then this could lead to increased virulence and lung damage in these individuals. Future work investigating the prevalence and association of these mutations with clinical outcomes will help test this theory.

Second, these data show that efflux pump inhibitors might have unexpected negative consequences on infections in people. Efflux pump inhibitors such as PAβN can inhibit efflux and potentiate other antibiotics that would normally be expelled through those pumps [27]. However, the experiments presented here show that inhibiting or deleting one efflux pump, MexEF-OprN, can have the unintended consequence of increasing rhamnolipid gene expression. Thus, inhibitors that affect MexEF-OprN may not be good candidates for use clinically. Instead, it would be more beneficial to identify more specific efflux pump inhibitors. For example, the MexAB-OprM-specific efflux pump inhibitor D13-9001 [39] could be used to block MexAB-OprM without inhibiting MexEF-OprN, and this could make *P. aeruginosa* more susceptible to other antibiotics and reduce virulence, since *mexAB* deletion mutants are less virulent than WT *P. aeruginosa*, as shown here and in other research [40]. Our data highlight the need to explore these types of precise approaches in future research using animal infection models.

### 3.7. Limitations of the Present Work

It is also important to acknowledge the limitations of this work. First, the strain PAO1-AzEvB8 that was the focus of the present study was not an actual clinical isolate and it is unclear how frequently mutants arise that have both *mexR* and *mexEF* mutations. As mentioned above, literature suggests that *mexR* mutations are very common in CF, and that clinical isolates can display varying degrees of *mexE* gene expression [20,22,24,33,36,37], so we feel that this study still has relevance to real-world *P. aeruginosa* infections.

The second primary limitation to this work is that the mouse model analyzed does not perfectly model the chronic infections that affect people with CF. One weakness is that this model does not capture the types of nutrient and biochemical properties present in CF that can affect *P. aeruginosa* antibiotic tolerance and virulence factor expression [41,42,43,44]. However, one strength to this model is that it does recapitulate the airway epithelial cell barrier and neutrophils present in CF infections that are susceptible to rhamnolipid-mediated toxicity [13,14]. To explore the effects of these mutations on other aspects of CF lung disease, future work could utilize chronic infection models in rodents or the more recently developed CF ferret and pig models [14,45,46,47,48].

One final limitation to this work is that the deletion mutants have not been complemented. Therefore, it is possible that the swarming and virulence phenotypes could be driven by the off-target effects of the generated mutations. We believe this is unlikely because we were able to reproduce identical phenotypes in multiple strains possessing related mutations. For example, the PAO1-AzEvB8 strain has both a *mexR* null mutation and *mexEF* deletion mutation, yet we only saw the swarming phenotype increased in the PAO1-AzEvB8 and Δ*mexEF* strains, not the Δ*mexR* or Δ*mexAB* strains. This suggests that the swarming phenotype in the PAO1-AzEvB8 strain is driven by its *mexEF* mutation. This was also supported by experiments with Tn mutants, where we observed increased swarming in *mexE, mexF,* and *oprN* Tn mutants, but not in a *mexR* Tn mutant. An additional caveat is that the clean deletion mutation in the PAO1 Δ*mexEF* strain was not generated in-frame with the *mexE* and *mexF* coding sequences, whereas the *mexR* and *mexAB* deletion constructs were generated in-frame with the original genes. Therefore, it is possible that a small peptide may be transcribed in the Δ*mexEF* strain that is out-of-frame (+1) relative to the *mexEF-oprN* coding sequences. This could have two consequences independent of the *mexEF* deletion. First, the Δ*mexEF* strain phenotypes could be caused by this novel peptide and not the *mexEF* deletion which was confirmed by whole genome sequencing. We think this is unlikely because the other phenotypes of this strain, including increased antibiotic susceptibilities to ciprofloxacin and chloramphenicol are consistent with previous studies of *mexE* mutants [26,35]. The other possibility is that these phenotypes could be caused by the effects of the *mexEF* deletion that prevent normal expression of *oprN*. Even if this were true, the phenotypes would still be consistent with the overall loss-of-function of the *mexEF-oprN* efflux pump, which is consistent with our conclusions. Long-term, we plan to perform complementation studies to tease out whether the swarming, rhamnolipid, and virulence phenotypes are due to loss-of-function of all three genes in the *mexEF-oprN* operon, or if phenotypes can be caused by mutation by each of the individual genes.

## 4. Materials and Methods

### 4.1. Bacterial Strains and Growth Conditions

Bacterial strains and plasmids are listed in Table S1. Mutant strains were derived from WT PAO1, which was obtained from Colin Manoil’s laboratory at the University of Washington [49]. Tn mutants were also obtained from Colin Manoil’s laboratory at the University of Washington and they were grown from freezer stock on Luria−Bertani (LB) agar with 10 μg/mL tetracycline [49]. All other strains were routinely grown at 37 °C on LB agar and in LB broth (cat# 244520 and 244620, Becton & Dickinson Co., Franklin Lakes, NJ, USA) unless otherwise specified.

### 4.2. Deletion Plasmid Construction

PAO1 strains carrying full deletions of *mexAB*, *mexEF*, or *mexR* genes listed in Table S1 were generated with a suicide plasmid as described previously [50]. For the Δ*mexAB* and Δ*mexR* strains*,* two PCR fragments were generated from chromosomal DNA for each construct using the following primer pairs up- and down-stream of each target for mutation; *mexAB* genes: mexAB-KO-UP-F and mexAB-KO-UP-R for the upstream *mexAB* fragment and mexAB-KO-DN-F and mexAB-KO-DN-R for the downstream *mexAB* fragment; and *mexR*: mexR-KO-UP-F and mexR-KO-UP-R for the upstream *mexR* fragment and mexR-KO-DN-F and mexR-KO-DN-R for the downstream *mexR* fragment. The suicide plasmid pEX18Gm [51] was prepared for assembly by restriction digest or PCR amplification with primers pEX18Gm-F and pEX18Gm-R (Supplementary Material Table S1). The two fragments for each construct were then assembled into the prepared vector pEX18Gm [51] using NEBuilder HiFi DNA Assembly Cloning Kit (cat# E5520, New England BioLabs, Ipswich, MA, USA) for the Δ*mexAB* mutant and the NEB Gibson Assembly Master Mix (cat# E2611L, New England BioLabs, Ipswich, MA, USA) for the Δ*mexR* mutant. Suicide plasmids were transformed into *E. coli* DH5α [52] competent cells (NEB cat# C2987H, New England BioLabs, Ipswich, MA, USA) according to manufacturer’s protocol, selected on LB agar with 10 μg/mL gentamicin (Gm). Plasmids were isolated using the NEB Monarch Plasmid Purification MiniPrep Kit (cat# T1010L, New England BioLabs, Ipswich, MA, USA) and verified by Sanger sequencing. For the Δ*mexEF* mutant construct, two sets of primers (oRP_21, oRP_22 and oRP_23, oRP_24) were designed to amplify 527 and 495 bp regions upstream and downstream, respectively, of the *mexEF* genes. These PCR fragments were assembled by splicing by overlap extension PCR and the deletion allele, containing attachment sites (*attB1*- and *attB2-*) for Gateway recombination, was integrated into the pDONRPEX18Gm vector via the BP Clonase reaction, as previously described [50]. The reaction mixture was transformed into *E. coli* DH5α by electroporation, and clones harboring the plasmid with the Δ*mexEF* allele were selected on LB agar with 10 μg/mL Gm and identified by colony PCR using M13 universal primers. This yielded plasmid pRP12 (pDONRPEX18Gm::Δ*mexEF*), which was sequence verified using M13 primers.

### 4.3. Transformation of P. aeruginosa Deletion Mutants

PAO1 deletion mutants were generated from WT *P. aeruginosa* PAO1 through two-step allelic exchange through either electroporation or mating, as described previously [50]. For electroporation of pEX18Gm::Δ*mexAB*, 1.5 mL of PAO1 or PAO1-AzEvB8 grown overnight in LB were centrifuged at 14,000× *g* for 3 m and washed twice with 300 mM sterile sucrose. Cells were then centrifuged at 14,000× *g* for 3 m and resuspended in 100 μL of 300 mM sterile sucrose. One microgram of pEX18Gm::Δ*mexAB* was then added to each cell suspension, and the bacterial suspensions were transformed by electroporation at 2.5 kv and incubated on LB with 30 μg/mL Gm at 37 °C overnight. For counter selection, isolated clones were streaked on low-salt LB containing 15% sucrose as published [50] and incubated at room temperature for 48 h. Gene deletions were confirmed by PCR using primers mexAB-KO-Chk-F and mexAB-KO-Chk-R (Table S1) and Sanger sequencing. For the Δ*mexR* strain, pEX18Gm::Δ*mexR* was transformed into chemically competent *E. coli* SM10(λ_pir_) [53] which were prepared using transformation and storage solution (TSS, LB broth with 10% (*w/v*) polyethylene glycol, 5% (*v/v*) dimethyl sulfoxide, and 0.5% (*w/v*) MgSO_4_·7H_2_O), as described previously [54] and selection on LB agar with 20 μg/mL Gm. *E. coli* SM10(λ_pir_) pEX18Gm::Δ*mexR* was mixed with PAO1, spotted onto an LB plate, and incubated overnight at 30 °C. Matings were collected and plated on VBMM agar with 60 μg/mL Gm to select for *P. aeruginosa* merodiploids and incubated overnight at 37 °C, as described [50]. Merodiploid colonies were streaked onto LB agar with 15% sucrose and incubated at 25 °C for 4 days. PAO1 Δ*mexR* strains were confirmed by PCR amplification using primers mexR-KO-UP-F and mexR-KO-DN-R (Table S1). To generate the PAO1 Δ*mexEF* strain, pDONRPEX18Gm::Δ*mexEF* was transformed into the donor *E. coli* S.17.1 (λ_pir_) strain. Biparental mating was used to introduce the suicide plasmid into the *P. aeruginosa* PAO1 recipient. Merodiploids resistant to sucrose but sensitive to Gm were isolated by counter-selection on no-salt-LB agar with 15% *w/v* sucrose after two days of incubation at 30 °C. Colony PCR was performed to identify Δ*mexEF* mutants and PCR products generated from the cloned mutant allele were sent for sequencing with oRP_27 and oRP_28 primers. Using this protocol, a 4397 bp fragment from the 4434 bp *mexEF* sequence was removed resulting in a frameshift mutation in the *mexEF-oprN* multidrug efflux operon, yielding strain RP05 (PAO1 Δ*mexEF*) (Table S1).

### 4.4. PAO1 Transformation with rhlA Reporter Plasmid

To quantify the expression of genes involved in rhamnolipid production, strains PAO1, PAO1-AvEvB8, PAO1 Δ*mexR*, PAO1 Δ*mexEF*, and PAO1 Δ*mexAB* were transformed with pYL122, a plasmid containing *rhlA*-*gfp* promoter fusion in a mini-CTX-*lacZ* backbone [55]. Following transformation, strains were maintained on LB plates with 100 μg/mL tetracycline. Clones were then tested using PCR with the primers pYL122-Chk-F and pYL122-Chk-R (Table S1).

### 4.5. DNA Extraction, Purification, and PCR

Plasmid DNA was prepared using Monarch Plasmid Miniprep Kit (cat# T1010, New England BioLabs, Ipswich, MA, USA). Genomic DNA was prepared using DNeasy Blood & Tissue Kit (cat# 69504, Qiagen, Hilden, Germany). When necessary, cDNA was purified using Monarch PCR & DNA Cleanup Kit (cat# T1030, New England BioLabs, Ipswich, MA, USA). PCR was performed using either KAPA HIFI 2X ready mix (cat# KK2602, KAPA Biosystems, Wilmington, MA, USA).

### 4.6. Swarming Assay

Swarming plates were created using 2.5% LB and 0.5% agar. For the assay, all strains were grown overnight for 16–20 h in LB broth without antibiotics, including Tn mutants. For each strain, 2 μL of the overnight broth was then placed at the center of a swarming plate and left to incubate at 37 °C for 24 h. The maximum swarming diameter was then measured and recorded for each strain.

### 4.7. Gradient Diffusion Antibiotic Susceptibility Testing

Aztreonam, ciprofloxacin, and chloramphenicol Etest strips were purchased from bioMérieux (cat# 501758, 412310, 412308, Durham, NC, USA) and antimicrobial susceptibility testing was performed with the following modifications to the manufacturer’s instructions. For aztreonam, a sterile swab was soaked in an overnight culture for each strain after growth for 18 h in LB broth and excess fluid was removed by pressing it against the inside wall of the test tube. For ciprofloxacin and chloramphenicol, colonies were picked directly from LB agar plates and diluted in sterile PBS to OD_600_ 0.15 (~1.5 × 10^8^ CFU/mL). Mueller Hinton agar plates were fully streaked 4 times with the swabs. After allowing the plates to dry, an Etest gradient strip was placed in the middle of the plates. Plates were incubated at 37 °C for 16–20 h.

### 4.8. rhlA GFP Reporter Assay

WT PAO1, PAO1 *attB::rhlA-gfp*, PAO1-AzEvB8, PAO1-AzEvB8 *attB::rhlA-gfp*, Δ*mexR*, Δ*mexR attB::rhlA-gfp*, Δ*mexEF*, Δ*mexEF attB::rhlA-gfp*, Δ*mexAB*, and Δ*mexAB attB::rhlA-gfp* strains were grown overnight for 16–20 h in 2 mL LB. The overnight cultures were then diluted to OD_600_ ~0.005 (~5 × 10^6^ CFU/mL). Two hundred microliters of the working dilutions were added in triplicate to a sterile black (clear bottom) 96-well plate. To prevent evaporation, 50 μL of mineral oil was added to each well. The plate was incubated for 24 h at 37 °C, shaking at 250 rpm, and absorbance at 600 nm and fluorescence with excitation at 488 nm and emission at 510 nm were read every hour for 24 h. For each strain, fluorescence was first normalized to the absorbance value. The background of each nontransformed strain was then subtracted from its pYL122-carrying counterpart. Gene expression was calculated as area under the curve using Prism GraphPad. For the efflux pump inhibitor assay, the *rhlA* reporter assay was performed as described above with the exception that the efflux pump inhibitor PAβN (Phe-Arg β-naphthylamide dihydrochloride, Sigma-Aldrich, Burlington, MA, USA, cat# P4157-25MG) was added to cultures to reach final concentrations of 0 μg/mL, 5 μg/mL, 10 μg/mL, and 25 μg/mL.

### 4.9. Murine Lung Infection

Experiments were approved by the Institutional Animal Care and Use Committee at Cedars-Sinai Medical Center under protocol IACUC008115. Strains were grown to mid-exponential phase, washed with sterile PBS, and diluted to 1 × 10^8^ CFU/mL in sterile PBS. Female C57BL/6 (Jackson Laboratories, Bar Harbor, ME, USA) 12-week-old mice (10 mice/group) were anesthetized using isoflurane. A 24-gauge angiocatheter was used to intubate the mice. Acute lung infections were performed by single intratracheal instillations of 5 × 10^6^ CFUs in 50 μL sterile PBS. During inoculation the mice were placed on a surgical board in the supine position. The front paws of each mouse were stretched outwards. A rubber band was placed around the two front incisors of the mouse to help keep their mouths open during the intubation procedure. After inoculation mice were kept in a clean cage under infrared lamp until full recovery. Mice were evaluated thrice per day to assess morbidity, and moribund mice were sacrificed with inhaled CO_2_. Surviving mice were euthanized 96 h post-infection. Survival curves were analyzed with log-rank tests as described below.

### 4.10. Genome Sequencing and Analysis

To verify mutations in engineered *P. aeruginosa* strains, DNA was isolated from strains PAO1, PAO1 Δ*mexAB*, PAO1-AzEvB8, PAO1-AzEvB8 Δ*mexAB*, and PAO1 Δ*mexEF* and subjected to whole genome sequencing. DNA isolation was performed using a DNeasy Blood and Tissue Kit (Qiagen, Hilden, Germany). DNA was submitted to the Microbial Genome Sequencing Center at the University of Pittsburgh where libraries were prepared and sequenced using an Illumina NextSeq platform. Sequencing reads were analyzed using Breseq [56], comparing reads for each strain to the *P. aeruginosa* PAO1 parent reference genome. Figure showing deletion in the *P. aeruginosa* PAO1-AzEvB8 strain was created using CLC Genomics Workbench Software. 

### 4.11. Statistics

Statistical analyses were performed using Prism GraphPad Software v9 and SAS. For swarming and *rhlA* reporter assays, one-way ANOVA tests were performed followed by Tukey’s multiple comparison test. For survival analyses, survival times were tested across strata with a log-rank test, with Bonferroni-adjusted *p*-values where >2 strata were compared.

## 5. Conclusions

Altogether, this work sheds important light on how antibiotic-resistance mutations can affect efflux pumps and subsequent *P. aeruginosa* virulence. Long-term, we believe that new strategies for treating efflux pump overexpression or deletion mutants should be pursued. It will be particularly important to study the effects of efflux pump mutations in the context of biofilms and aggregates, both in vitro and in vivo, because this is the predominant bacterial mode of growth during CF infections. Additionally, we plan to explore how *mexR* mutations lead to increased virulence, since this mechanism still remains mysterious.

## Data Availability

Genome sequencing data are available through the National Center for Biotechnology Information (NCBI) Sequence Read Archive through BioProject accession number: PRJNA766087, http://www.ncbi.nlm.nih.gov/bioproject/766087.

## Supplementary Materials

The following are available online at https://www.mdpi.com/article/10.3390/antibiotics10101164/s1, Table S1: Strains and primers used in this study.
