# Supplementary material for: Pseudomonas aeruginosa mexR and mexEF Antibiotic Efflux Pump Variants Exhibit Increased Virulence
Source: Antibiotics (Basel). 2021 Sep 25;10(10):1164. doi: 10.3390/antibiotics10101164 (PMC8532662; doi:10.3390/antibiotics10101164)
Supplement: Supplementary file 1 [file antibiotics-10-01164-s001.zip › antibiotics-1383512-supplementary.pdf]

Supplemental Materials

Table S1. Strains, plasmids, and primers used in this study.

Strain or Plasmid	Relevant Characteristics	Reference/Source
PAO1	Wildtype *P. aeruginosa* strain	Held et al., 2012 [49]
PAO1-AzEvB8	PAO1 strain with *mexR* E118* and 20kb deletion (*mexF-antC*) mutations	Jorth *et al.*, 2017 [20]
PAO1-AzEvB8 Δ*mexAB*	PAO1-AzEvB8 deletion of *mexAB* genes	This study
PAO1 Δ*mexR*	PAO1 strain with a complete deletion of the *mexR* gene	This study
PAO1 Δ*mexAB*	PAO1 strain with a complete deletion of the *mexA* and *mexB* genes	This study
PAO1 Δ*mexEF* (RP05)	PAO1 strain with a complete deletion of the *mexE* and *mexF* genes	This study
PAO1 *mexE* Tn mutant	PAO1 Two-Allele transposon library strain PW5180; mexEH04:: ISphoA/hah	Held *et al.*, 2012 [49]
PAO1 *mexF* Tn mutant	PAO1 Two-Allele transposon library strain PW5184; mexFG01:: ISphoA/hah	Held *et al.*, 2012 [49]
PAO1 *oprN* Tn mutant	PAO1 Two-Allele transposon library strain PW5186; oprNC04:: ISphoA/hah	Held *et al.*, 2012 [49]
PAO1 *mexR* Tn mutant	PAO1 Two-Allele transposon library strain PW9404 mexRG01: ISphoA/hah	Held *et al.*, 2012 [49]
PAO1 PA3303 Tn mutant	PAO1 Two-Allele transposon library strain PW6550; PA3303H03: ISphoA/hah	Held *et al.*, 2012 [49]
PAO1-AzEvB8 *rhlA*::GFP	PAO1-AzEvB8 with *rhlA* GFP transcriptional reporter at *attB* site	This study
PAO1 Δ*mexR attB*::*rhlA-gfp*	PAO1 Δ*mexR* with *rhlA* GFP transcriptional reporter at *attB* site	This study
PAO1 Δ*mexAB attB::rhlA-gfp*	PAO1 Δ*mexAB* with *rhlA* GFP transcriptional reporter at *attB* site	This study
PAO1 Δ*mexEF attB::rhlA-gfp*	PAO1 Δ*mexEF* with *rhlA* GFP transcriptional reporter at *attB* site	This study
E. coli DH5α	*E. coli* strain used for cloning	Taylor et al., 1993 [52]
E. coli DH5α pEX18Gm	*E. coli* cloning strain used to propagate pEX18Gm	Gift from Pradeep K. Singh Lab
E. coli DH5α pEX18Gm::*ΔmexAB*	Suicide plasmid carrying Δ*mexAB* deletion construct	This study
E. coli DH5α pEX18Gm::*ΔmexEF*	Suicide plasmid carrying Δ*mexEF* deletion construct	This study
E. coli DH5α pEX18Gm::*ΔmexR*	Suicide plasmid carrying Δ*mexR* deletion construct	This study
E. coli SM10(λpir)	*E. coli* mating strain	Chung et al., 1989 [54]

E. coli SM10(λpir) pEX18Gm::*ΔmexR*	*E. coli* mating strain used to transform *pEX18Gm::ΔmexR* into *P. aeruginosa*	This study
E. coli SM10(λpir) pYL122	E. coli mating strain carrying pYL122 with *rhlA-gfp* promoter fusion	Lequette and Greenberg, 2005 [55]
pYL122	Plasmid containing a *rhlA-gfp* promoter fusion in a mini-CTX-*lacZ* backbone	Lequette and Greenberg, 2005 [55]
E. coli DH5α pEX18Gm::*ΔmexEF* (RP29)	pUC18-miniTn7T2.1-Gm-GW bearing Δ*mexEF*, Gm^r	This study
E. coli S.17.1 (λ_{pir}) pEX18Gm::*ΔmexEF* (RP12)	pUC18-miniTn7T2.1-Gm-GW bearing Δ*mexEF*, Gm^r	This study
pEX18Gm	Suicide plasmid for generating *P. aeruginosa* mutants	Hoang et al., 1998 [51]
pEX18Gm::Δ*mexAB*	Suicide plasmid carrying Δ*mexAB* deletion construct	This study
pDONRPEX18Gm::Δ*mexEF* (pRP12)	Suicide plasmid carrying Δ*mexEF* deletion construct	This study
pEX18Gm::Δ*mexR*	Suicide plasmid carrying Δ*mexR* deletion construct	This study

**Primers**	**Sequence 5'-3'**
mexAB-KO-UP-F	ACAAGCACCTGCGCAGCG
mexAB-KO-UP-R	CATAGCGTTGTCCTCATGAGCG
mexAB-KO-DN-F	GAAAAGGGGCAATGATATGAAAC
mexAB-KO-DN-R	GTCGAACAGGCCGGACAG
mexAB-KO-Chk-F	GTGTACTGGTTCCGGCCCT
mexAB-KO-Chk-R	CAGCCGGACAGAACGACAG
pEX18Gm-F	CTGGCCGTCGTTTTACAAC
pEX18Gm-R	TCATGGTCATAGCTGTTTC
mexR-KO-UP-F	gtaaaacgacggccagTCTGCGCTTCCAGGGTCAC
mexR-KO-UP-R	aatatcctcGGGGTAGTTCATTGGTTTGGC
mexR-KO-DN-F	actaccccGAGGATATTTAAGAACATTCTTTTCGAAGC
mexR-KO-DN-R	aacagctatgaccatgaCACCAACAGCGTGGACAC

oRP_21(PAO1_mexEFupF01)	GGG GAC AAG TTT GTA CAA AAA AGC AGG CTA CGC AAG CGC AAG GTG GTC C
oRP_22(PAO1_mexEFupR01)	***CTC TGG CAG GCC TTT GTC GTT GGC G***GG TAG CGC CAG GAG AAG TG
oRP_23(PAO1_mexEFdownF01)	CGC CAA CGA CAA AGG CCT G
oRP_24(PAO1_mexEFdownR01)	GGG GAC CAC TTT GTA CAA GAA AGC TGG GTA CTC CAG CTG ACG GCG GAT G
oRP_27(PAO1_mexEFseqF01)	CAA GCG CAA GGT GGT CCT G
oRP_28(PAO1_mexEFseqR01)	CGA ACA GGT CAA GCT CCC AG
pYL122-chk-F	TCAGCGGGGTTCAGTACG
pYL122-chk-R	CGATAGAGTTTGACAGTGTGTTGC

Note *attB*1 and *attB*2 recombination sites are highlighted in grey
